# Supplementary material for: Spatial Variation in Stream-Water Bacterial Communities Across Sites Differing in Wild Chinese Giant Salamander Detection Frequency
Source: Biology (Basel). 2026 Sep 18;15(18):1647. doi: 10.3390/biology15181647 (PMC13604818; doi:10.3390/biology15181647)
Supplement: Supplementary file 1 [file biology-15-01647-s001.zip › Data Related to “Spatial Variation in Stream-Water Bacterial Communities across Sites Differing in Wild Chinese Giant Salamander Detection Frequency”/Microbial Data/result/03.TaxonVisual/03.TaxonVisual_ReadMe.pdf]

扩增子结果文件补充说明（03）

2023年8月28日

03.TaxonVisual 【注释结果可视化】

1. barplot【物种相对丰度柱形图展示】

1.1 sample/group1/... 【样本及分组的物种相对丰度柱形图】

1.2 top10.*.*.barplot.{xls,png,svg} 【各（样本，组）门纲目科属种（p,c,o,f,g,s）水平上的 top10 物种相对丰度柱形图】

1.3 top30.*.g.barplot.{xls,png,svg} 【各（样本，组）属(g)水平的top30物种相对丰度柱形图】

2. heatmap【物种注释聚类热图】

2.1 sample/group1/... 【样本及分组的物种注释聚类热图】

2.2 heatmap.*.*. {pdf,png,txt} 【各（样本，组）门纲目科属种（p,c,o,f,g,s）水平上的物种聚类热图】

3. tree100 【属水平top100物种进化树圈图】

3.1 sample/group1/... 【样本及分组的属水平top100物种进化树圈图】

3.2 genus_100.tree 【属水平top100物种进化树】

3.3genus_100.tree.{png,svg} 【属水平top100物种进化树圈图】

4. venn【基于 OTU/ASV 的Venn图】

4.1 sample/group1/... 【样本及分组基于 OTU/ASV 的Venn图】

4.2 * 【进行venn图分析的（样本，组）名字，以-连接，例A-B】

4.3 *_*.txt 【（样本，组）特有的OTU/ASV，例 A-B_A.txt(A特有)】

4.4 *_*:*.txt 【（样本，组）共有的OTU/ASV，例 A-B_A：B.txt(A,B共有)】

4.5 *. {png,svg} 【（样本，组）Venn图展示】

5. flower【基于OTU/ASV 的花瓣图】

5.1 sample/group1/... 【样本及分组基于 OTU/ASV 的花瓣图】

5.2 * 【进行花瓣图分析的（样本，组）名字，以-连接，例A-B-C-D-E-F】

5.3 *_all-core.xls【（样本，组）共有的OTU/ASV，例 A-B-C-D-E-F_all-core.xls(共有 OTU/ASV)】

5.4 *_*.xls 【每个（样本，组）特有的OTU/ASV，例 A-B-C-D-E-F_A.xls(A特有)】

5.5 *. {xls,png,svg} 【（样本，组）花瓣图作图数据及可视化展示】

6. ternary 【三元相图分析结果】

6.1 sample/group1/... 【样本及分组三元相图分析结果】

6.2 */*.ternary.{jpeg,pdf} 【各（样本，组）门纲目科属种（p,c,o,f,g,s）水平上的三元相图可视化展示】
